# Model of hindlimb unloading in adult female rats: Characterizing bone physicochemical, microstructural, and biomechanical properties

**DOI:** 10.1371/journal.pone.0189121

**Published:** 2017-12-11

**Authors:** Melise Jacon Peres-Ueno, Camila Tami Stringhetta-Garcia, Robson Chacon Castoldi, Guilherme Akio Tamura Ozaki, Antônio Hernandes Chaves-Neto, Rita Cássia Menegati Dornelles, Mário Jefferson Quirino Louzada

**Affiliations:** 1 Programa de Pós-Graduação Multicêntrico em Ciências Fisiológicas, SBFis, São Paulo State University (UNESP), Araçatuba, São Paulo, Brazil; 2 Department of Orthopedics and Traumatology, Faculty of Medical Sciences, UNICAMP-Univ. Estadual de Campinas, Campinas, São Paulo, Brazil; 3 Department of Basic Sciences, School of Dentistry, São Paulo State University (UNESP), Araçatuba, São Paulo, Brazil; University of California Davis, UNITED STATES

## Abstract

Prolonged bedrest and microgravity induce alterations to bone, leading to bone fragility and compromising the quality of life. In this study, we characterized the physicochemical changes, microstructure, and biomechanics of the femurs of female adult rats in response to hindlimb unloading for 21 days. Twenty 6-month-old Wistar female rats were distributed into control (CON) and hindlimb unloading (HLU) groups. Analysis the *in vivo* bone mineral density (BMD) by dual energy x-ray absorptiometry (DXA) from the femurs was performed at the beginning and end of the experiment; plasma levels of calcium, phosphorus, and alkaline phosphatase, tartrate-resistant acid phosphatase activity, assessed by spectrophotometry, and estradiol, measured by enzyme-linked immunosorbent assay, was performed after the experiment. We evaluated changes in the trabecular and cortical structure of the femur, after disuse, by micro-computed tomography with high resolution, for analysis of cortical porosity, Raman spectroscopy to measure the amount of physicochemical properties, and the biomechanical test to estimate the changes in biomechanical properties. Our results demonstrated that, after 21 days, HLU animals had decreased femoral BMD, deteriorated bone microarchitecture, particularly in the cortical compartment, with changes in the physicochemical properties and porosity, and reduced deformation capacity of the bone and resistance to the bone stresses. Nevertheless, this study showed the critical role of mechanical stimulation in maintaining the structure of the skeleton in female adults and that disuse, even for a few days, leads to microscopic changes in the structure of the bone matrix, which increases the risk of fracture.

## Introduction

Mechanical stimuli are critical to ensure the performance of the human skeleton, since the mechanical load results in adjustments to the size and shape of bone, increasing its resistance [1]. Mechanical removal, in which patients have some form of mobility loss or reduced function, as in patients at rest, immobilized, bedridden, or in a permanent vegetative state, and as with astronauts, triggers reduction in strength and bone mass [2–5] and increases the incidence of osteoporosis and fracture risk. The bones’ capacity to resist rupture depends not only on the amount of mineral tissue, but also on the microarchitecture, geometry, and quality of the physical and chemical composition of the bone tissue [6, 7]. The changes in the microstructure of cortical tissue, such as cortical porosity (Ct.Po%), are important determinants of the risk of fracture, regardless of bone mineral density (BMD),since they increase the fragility of bone [8, 9]. Changes in the mineralization rate, type B carbonate substitution, and crystallinity are directly related to the quality and mechanical competence of bone tissue [7], and may be additional risk factors for fracture [10].

Because of the limitations found in studies with humans, studies in animal models are used routinely to evaluate bone tissue. Hindlimb unloading (HLU) is a well-established method used to simulate the effects of microgravity or prolonged rest on the skeletal system [11–14]. Despite its widespread use, there are few studies involving this method in adult females, unlike the studies seeking to understand the pathophysiology of disuse in young animals and male models, of which there are more [15, 16]. Therefore, to understand the impact of disuse related bone loss in adult female humans and animals, it is important to characterize the bone quality encompassing factors, such as cortical and trabecular bone microarchitecture, and changes in bone mineral and matrix tissue properties, without the influence of reduction of plasma estrogen concentration.

No published studies have yet quantified and measured the amount of the chemical substances present in the bone tissue after HLU or any method that induces disuse. Furthermore, few studies have evaluated the Ct.Po (%) after disuse with this experimental model of female adults after 21 days of disuse. Therefore, the aim of this study was to characterize the physical and chemical changes, and microstructural and biomechanics of the femurs of 6-month old Wistar female rats in response to HLU for 21 days. For this, we used micro-computed tomography (micro-CT) to evaluate the changes in the trabecular and cortical structure, micro-CT with high resolution, for analysis of Ct.Po (%), Raman spectroscopy to measure the amount of physicochemical properties, and the biomechanical test to estimate the changes in biomechanical properties.

We hypothesized that disuse, induced by HLU over 21days in female Wistar rats, would cause reductions in bone density and biomechanics deterioration, which may be related to alterations of the bone microstructure and changes in physicochemical properties.

## Materials and methods

### Animals

Animal use and experimental procedures were approved by the Ethics Committee on Animal Use of the School of Dentistry, Araçatuba, São Paulo, Brazil (protocol number 01037–2013).

Twenty female Wistar rats, aged 5–6 months were housed at 22°C (±2°C) under a 12:12 hour light: dark cycle, in common cages, and were fed a diet of the same quality and manufacturer (Presence^®^-Ratos e Camundongos, Paulínia, SP, Brazil/12-13g/kg calcium), and water *ad libitum*. The general health of the rats was monitored daily and the estrous cycle was checked during the 5th month of life of the rats for evidence of regular estrous cycles, in accordance with the Long and Evans protocol [17]. At 6-months of age, the animals were randomized into two groups: control group (CON) and HLU group with ten animal each group.

### HLU

HLU was conducted when the rats were 6-months of age, for 21 days, as proposed by Morey-Holton and Globus [18], with modifications to the protocol, using adhesive foam (Reston), which was placed from one side to the other on the tail and fixed with tape (S1 Fig). The animals did not need to be anesthetized. After this procedure, was attached to a clip and hung in a threaded cylindrical bar with adjustable height on the top of the cage compartment. Thus, it allowed enough support of the forelimbs for the animals to get around and feed themselves. Two metal blocks were placed at the ends of the support bar, and were adjusted so as not to allow the animal to make contact with the walls of the cage. Water was provided by a cooler placed at a suitable height on the wall of the joint cage and feed was placed on the floor. The animals were suspended for 21 days and euthanasia of all experimental animals was performed the day after that with anesthetic overdose.

### Biochemical assay

Blood collection was performed at the end of the experiment (at 6 months) by cardiac puncture [19]. The blood was centrifuged (3.000 rpm;15 minutes; 4°C), plasma was fractionated and stored in a -80°C freezer. The samples were submitted for biochemical analysis using the spectrophotometric method to estimate the concentration of calcium (Calcium Liquiform, LabtestDiagnostica SA, MG, Brazil), phosphorus [20] (Phosphorus UV Liquiform, LabtestDiagnostica SA, MG, Brazil), total alkaline phosphatase [21] (Alkaline Phosphatase, LabtestDiagnostica SA, MG, Brazil), which were used as markers of bone formation, and tartrate-resistant acid phosphatase (TRAP) activity (Sigma-Aldrich, St. Louis, MO) [22, 23] which was used as a marker of osteoclast activity. Determination of plasma concentration of estradiol (IBL International GmbH, Hamburg, Germany) was performed using an enzyme-linked immunosorbent assay by solid phase and the detectable minimum dose of 0.28 pg/mL. All samples were measured in the same assay and with the same standard error of mean to avoid inter-assay variability.

### *In vivo* dual-energy X-ray absorptiometry (DXA)

The animals were anesthetized with ketamine hydrochloride (Vetaset-Fort Dodge/50 mg/kg, i.p.) and xylazine (Coopazine-Coopers Brazil Ltda/25 mg/kg, i.p.) to perform the *in vivo* bone densitometry of the femurs at the beginning and end of the experiment. After anesthesia, the animals were placed individually on the densitometer and were subjected to analysis of the total area (cm^2^), bone mineral content (g), and areal BMD) (g/cm^2^) measured in the whole femur by DXA (Lunar^®^ DPX-Alpha WI, USA) with special software for small animals, coupled to a computer.

### *Ex vivo* measurement of microarchitecture

After euthanasia, the femurs were immediately stored in saline at -20°C. Twenty-four hours prior to testing, the bones were transferred to a temperature of 7°C and then kept at room temperature until the end of the test. We performed non-destructive three-dimensional evaluations of the femur bones’architecture, with a micro-CT Skyscan 1174 (Skyscan, Aartselaar, Belgium; FINEP/CT-INFRA 01.12.0530.00-01/2011), conducted with an aluminum filter 0.5mm, 185° rotation, 0.4° step, 50kV, and 800 mA of configuration with resolution of 16 μm. The region of interest of the trabecular bone was manually interpolated and 50 slices in the region, comprising the head, neck and greater trochanter of the femur, without cortical bone, were analyzed, 150 slices below the first bone image. In the cortical bone of the femur, 100 slices of the femoral midshaft were analyzed, 750 slices below the first bone image.

The images were reconstructed using NRecon software (SkyScan, Leuven, Belgium) and the CTAn v.1.5.0 software (Skyscan) was used for three-dimensional (3D) analysis. The parameters analyzed for the trabecular bone were percentage of bone volume (bone tissue volume divided by total volume) (BV/TV) (%), trabecular thickness (Tb.Th) (mm), number of trabeculae per millimeter of tissue (Tb.N) (mm^-1^), trabecular separation (Tb.Sp) (mm), and structure model index (SMI), according to standard procedures [24]. For the cortical bone in the diaphysis of the femur, the parameters analyzed were total volume (Tt.V) (mm^3^), cortical bone volume (Ct.V) (mm^3^), medullary volume (Ma.V) (mm^3^), cortical volume fraction (Ct.V/Tt.V) (%), and average cortical thickness (Ct.Th) (mm).

For analyzing the Ct.Po (%), the femurs were scanned with the micro-CT Skyscan 1272 (Skyscan, Aartselaar, Belgium), conducted with an aluminum filter, 0.5mm, 185° rotation, 0.5° step, 70kV and 142 mA of configuration with a resolution of 6 μm. Ct.Po (%), pore number (Po.N) (n), and total pore volume (Po.V) (mm^3^) were analyzed at the femoral midshaft.

### Raman microspectroscopy

To determine the composition and relative intensities of the mineral and matrix in the midshaft of the right femur, Raman microspectroscopy “micro-Raman (Renishaw) in-Via model surface-enhanced Raman scattering” was used, with a laser wavelength of 633 nm, microwatts of power at the sample order (μW), and the grating with1800 lines per mm. The exposure time was set at 10 seconds and the accumulation number was set to five. Optical microscopy was performedwithan optical microscope Leica (DMLM series), coupled to the spectrograph and 50x objective, which is able to provide a spatial resolution of the order 1.00 μm^2^, Peltier CCD detector (cooled to -70°C) and xyz motorized stage (stepper motor—0.10 uM), in which samples were placed. Three spectra were collected from each sample. The ratios of peak intensities were used to calculate the mineral-to-collagen ratio (ν1PO4/Amide I) and Type B carbonate substitution (CO3/ν1PO4), in which ν1PO4, Amide I, and CO3 occurred at 959–960 cm^−1^, 1665–1669 cm^−1^, and 1070–1077 cm^−1^, respectively. Crystallinity was determined as the inverse of the full-width at half maximum, in which the maximum was the normalized peak intensity (one) relative to the baseline (zero) of the ν1PO4 peak [10].

### Biomechanical testing

The femurs on the right side of all animals were placed in the mechanical universal testing machine (DL 3000, EMIC^®^, SP, Brazil). The load was applied at a speed of 2 mm/min and with a of force 2000N, to determine the maximum load (N) and subsequent determination of bone stiffness (N/mm) and absorbed energy to maximum load (mJ), calculated from the curve obtained in the test [25]. The results were recorded in the computer system within the equipment itself, which provides the values of Force x deformation. For the calculation of the stiffness, part of the force-deformation curve relative to the elastic phase of the assay was determined. The measurement of the energy absorbed by the bone was obtained by the area under the force-strain curve to the point of maximum strength.

The tests used were:

1. Three-points bending testing: The femurs were supported by two supports, with 20 mm between the supports. The force was applied to the bone at a third point at the geometric mean between the two supports [25].
2. Biomechanical compression bending testing: For analysis of maximum load, stiffness, and energy in the femoral neck region, the femur was placed in the metal apparatus and maintained fixed vertically (long axis). The compression force was applied in the area of the femoral head, for which the vector line of action force is parallel to the long axis of the femur, causing bending motion in the head and femoral neck [26].

### Statistical analysis

Data were expressed as means ± standard deviation (SD). The normality of the data was analyzed with the Shapiro-Wilk normality test. For intra-group analysis at the beginning and end of the experimental period, data were submitted to a paired t-test. For comparison between groups at the end of the experiment, data were submitted to an unpaired t-test. The computer program GraphPadInstat^®^, version 6.0 was used. The significance level was 5% for all analyses.

## Results

### Animal characteristics

**Fig 1:**
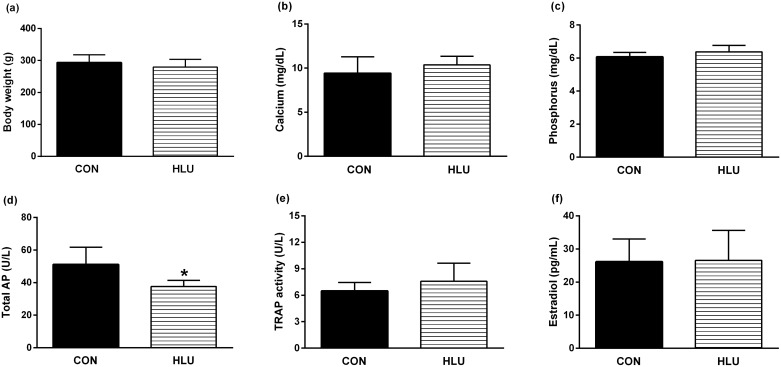
Effects of hindlimb unloading on body mass and biochemical parameters. Body weight (a), calcium (b), phosphorus (c), alkaline phosphatase (AP) (d), tartrate-resistant acid phosphatase (TRAP) activity (e), and estradiol plasma concentration (f) of control female rats (CON) and hindlimb unloading female rats (HLU). Statistical analysis was performed with Mann Whitney test (*P* < 0.05) to analyze the effect of hindlimb unloading (HLU). Animals number: 10/group. Abbreviations: AP = alkaline phosphatase; TRAP = tartrate-resistant acid phosphatase.

After the 21^st^ day of disuse, there were no significant effects on body weight, calcium, phosphorus, TRAP activity, and estradiol plasma concentration in the HLU group, compared with the CON group (Fig 1a–1f). The activity of alkaline phosphatase in the HLU rats was significantly lower compared to that in the CON group (*P* = 0.043) (Fig 1d).

### Bone densitometry

**Fig 2:**
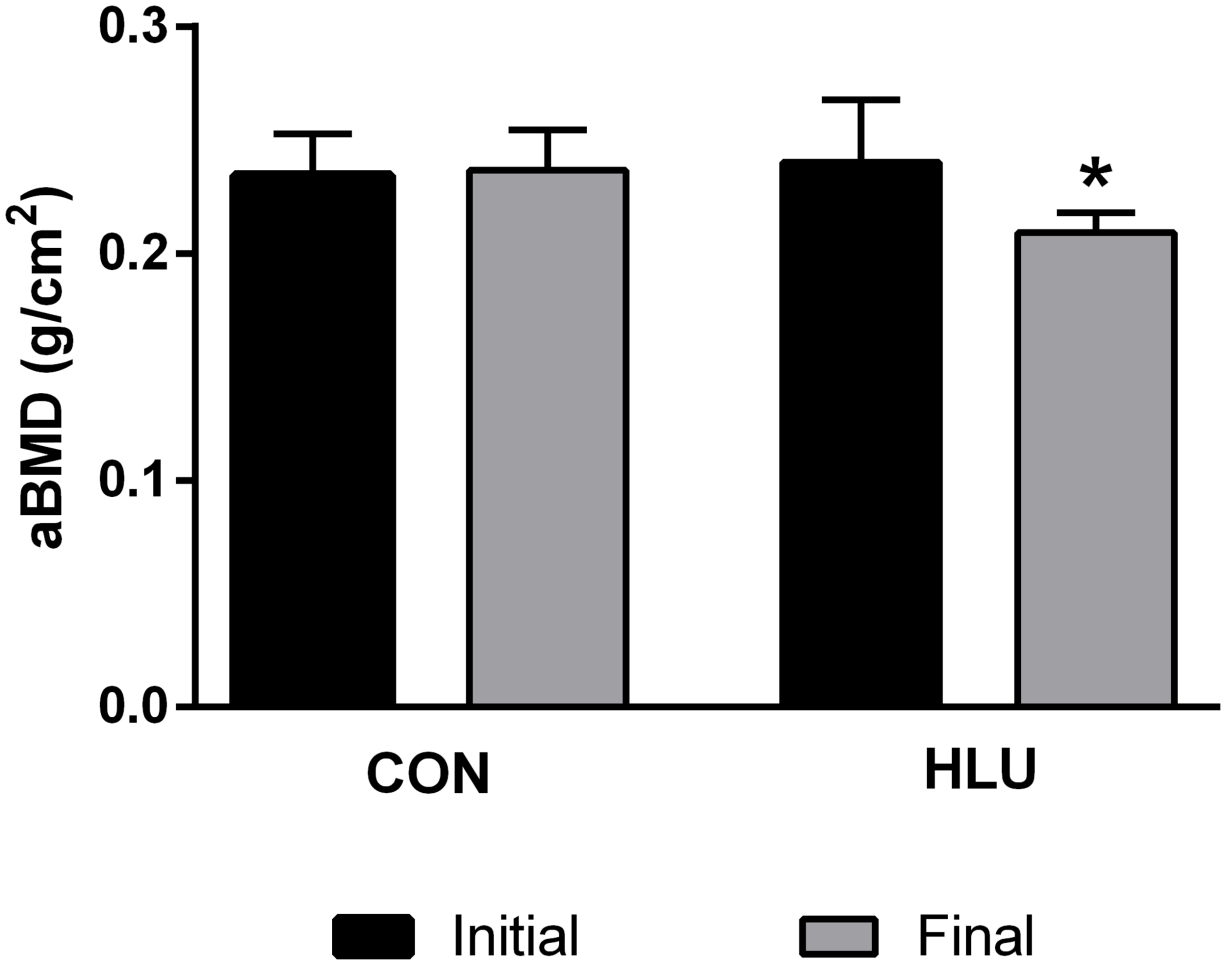
*In vivo* areal bone mineral density. Analysis of the areal bone mineral densitometry (aBMD) by dual-energy X-ray absorptiometry (DXA) of the femurs of female rats with 6-months of age (initial) and after 21 days (final). The control rats group (CON) moved freely through the box during the experimental period and the hindlimb unloading rats group (HLU) were suspended for 21 days. Each column represents mean ± standard deviation (SD). Statistical analysis was performed with Wilcoxon test for intra-group analysis at the beginning and end of the experimental period (*P* < 0.05) and Mann Whitney test to compare the HLU group to the CON group * (*P* < 0.05). Animals number = 10/group.

The aBMD femurs analysis, whether being the animal subject to hindlimb unloading or not, at 6-months of age female rats (initial) and after 21 days (final), was not changed between control animals at initial and final aBMD values (Fig 2). However, in animals of the HLU group, the aBMD after 21 days of disuse decreased in the femurs of this animals when compared with the aBMD at the beginning of the experiment (*P* = 0.007). Furthermore, when comparing the final aBMD between the groups, there were no significant differences (*P* > 0.05).

### Bone microarchitecture

**Fig 3:**
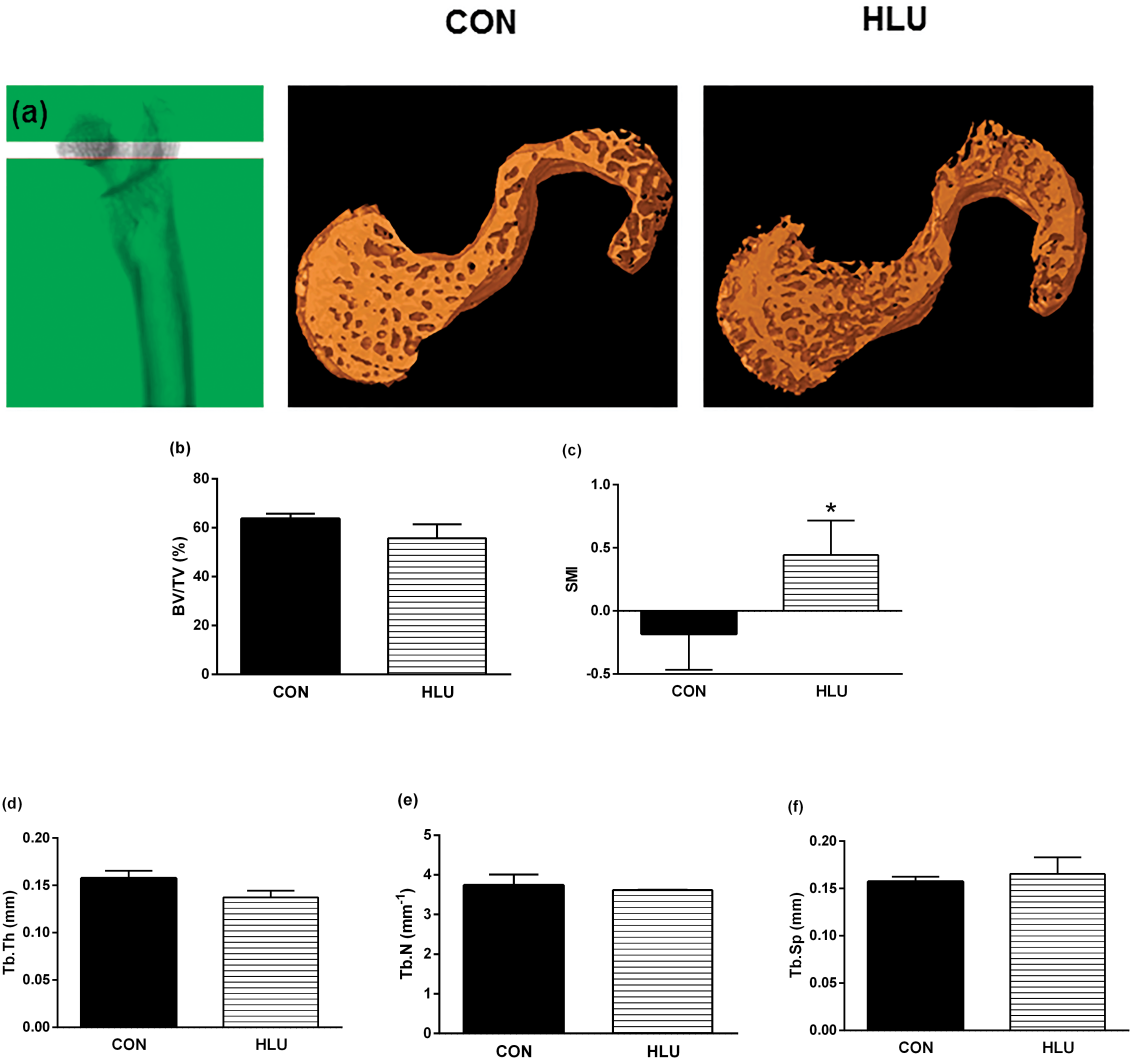
*Ex vivo* measurement of trabecular microarchitecture. Scanned image of the region of interest of the trabecular femoral neck (a). The 3D Reconstruction shows a trabecular bone in the femoral neck (50 slices) of the control (CON) group *(on the left)* and the hindlimb unloading (HLU) group *(on the right)* after 21 days of hind limb suspension. *Ex vivo* trabecular bone microarchitecture: bone tissue volume divided by total volume (BV/TV) (b), structure model index (SMI) (c), trabecular thickness (Tb.Th) (d), number of trabeculae per millimeter of tissue (Tb.N) (e), trabecular separation (Tb.Sp) (f), data of the femoral neck was assessed by micro-computed tomography (micro-CT). Each column represents mean ± standard deviation (SD). Statistical analysis was performed with the Mann Whitney test to compare the HLU group to the CON group * (*P*< 0.05).

**Fig 4:**
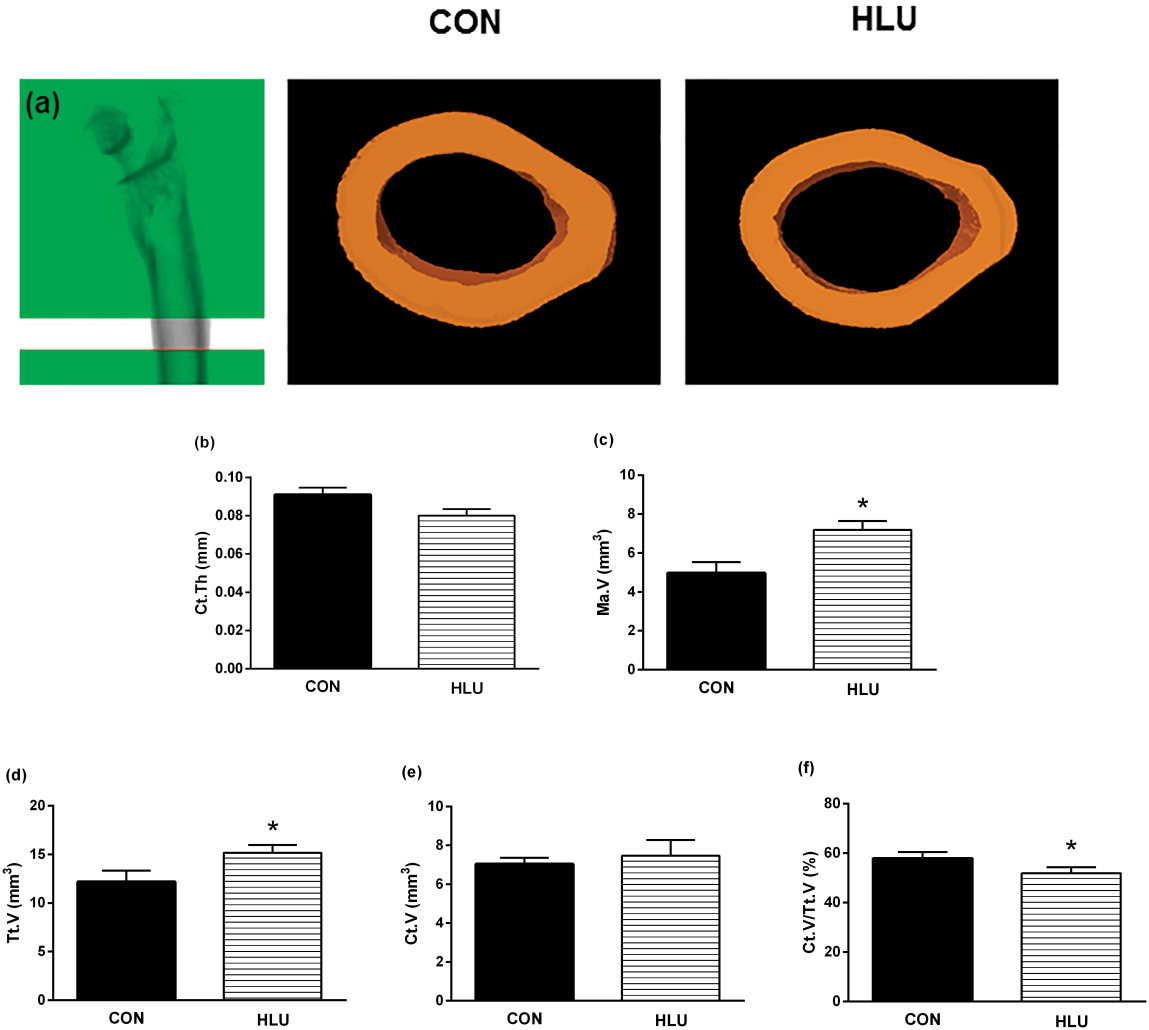
*Ex vivo* measurement of cortical microarchitecture. Scanned image of the cortical bone, with dimensions of the region of interest of the femoral midshaft (a). The 3D Reconstruction shows cortical bone in the femoral midshaft (100 slices) of the control (CON) *(on the left)* and the hindlimb unloading (HLU) *(on the right)*. *Ex vivo* cortical bone microarchitecture: cortical thickness (Ct.Th) (b), medullary volume (Ma.V) (c), total volume (Tt.V) (d), cortical bone volume (Ct.V) (e) and Ct.V/Tt.V (f) data of the femoral midshaft assessed by micro-computed tomography (micro-CT). Each column represents the mean ± standard deviation (SD). Statistical analysis was performed with the Mann Whitney test to compare the HLU group to the CON group * (*P*< 0.05).

The representative 3D reconstructed micro-CT images of the trabecular femoral neck bone and cortical bone microarchitecture of the femoral midshaft from each group are shown in Figs 3 and 4, respectively. Following the disuse period, almost no statistically significant changes in trabecular microstructure were detected in the female femoral neck. There were no significant differences between CON and HLU on BV/TV, Tb.Th, Tb.N and Tb.Sp (*P* >0.05) of the femoral microstructure (Fig 3b, 3d, 3e and 3f). However, the SMI (Fig 3c) of the HLU group was significantly higher compared with CON group (*P* = 0.031).

**Fig 5:**
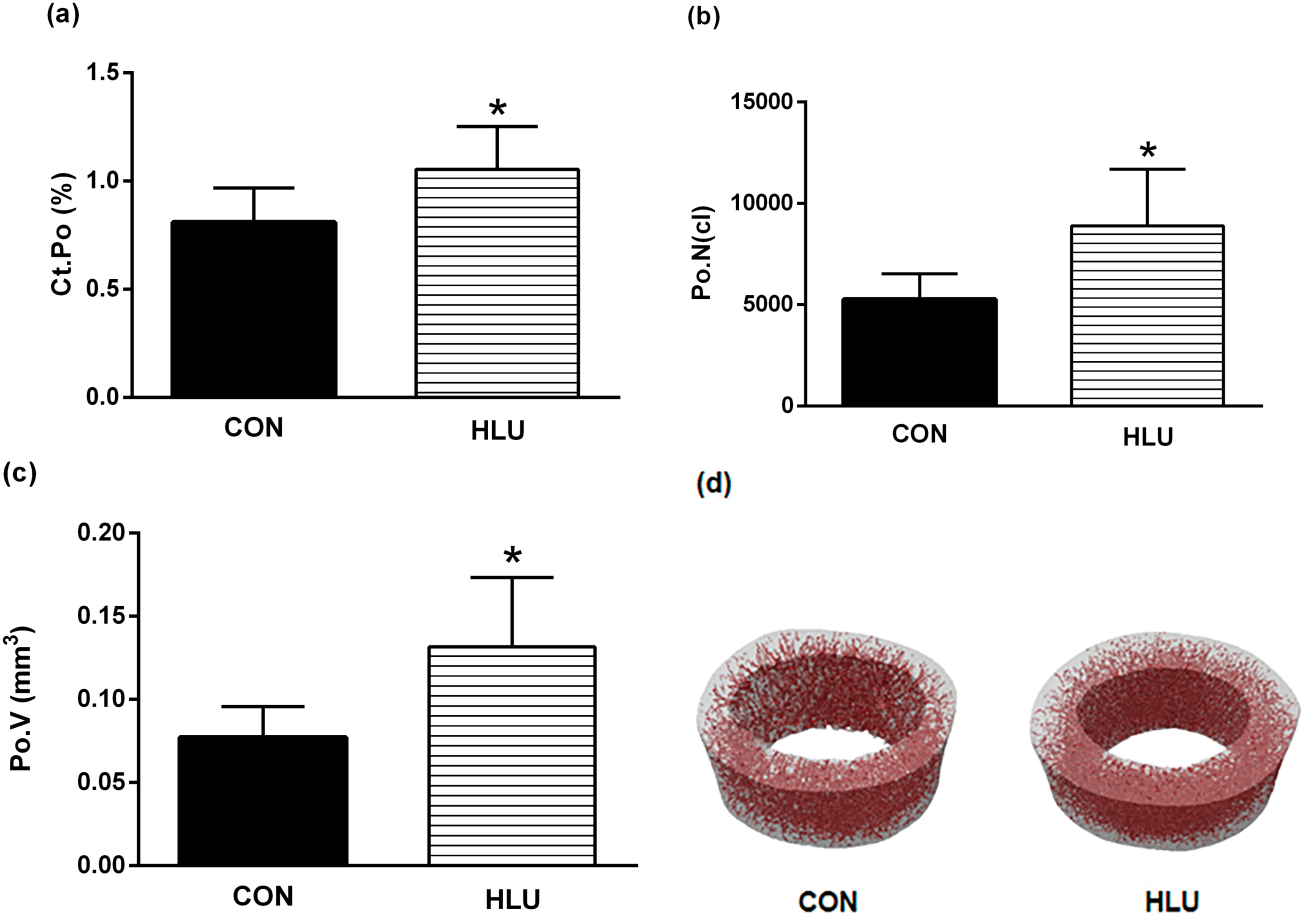
*Ex vivo* cortical porosity. Cortical porosity (Ct.Po; %) (a), pore number (Po.N; n) (b), and total pore volume (Po.V; mm^3^) (c), data of the femoral midshaft assessed by micro-computed tomography (micro-CT). Each column represents mean ± standard deviation (SD). Statistical analysis was performed with Mann Whitney test to compare the hindlimb unloading (HLU) group to the control (CON) group * (*P*< 0.05). The 3D reconstruction (d) shows the porosity *(in red)* in the femoral midshaft (100 slices) of the CON group *(on the left)* and the HLU group *(on the right)*.

Results from the cortical bone analysis demonstrated significant changes in the microarchitecture of the femoral midshaft as result of disuse (Fig 4). Regarding bone microarchitecture, the greatest changes following the disuse period were observed in the cortical region. Animals in the HLU group had significantly higher Tt.V (*P* = 0.015) and Ma.V (*P* = 0.028) of cortical bone compared with the CON group, and lower Ct.V/Tt.V (*P* = 0.028). There were no significant differences between the CON and HLU groups of the Ct.Th (*P* = 0.114). The difference between the CON group and HLU group for Ct.Po (%) was remarkable. Fig 5 demonstrates the visibly increased porosity in the femoral midshaft scan region after the 21-day disuse period. The porosity is represented by dark red shading and the bone by off-white coloring (Fig 5d). The disuse period resulted in a significant increase in Ct.Po (%), Po.N (n), and Po.V (mm^3^) at the affected site (*P* < 0.05).

### Bone biomechanical strength

**Fig 6:**
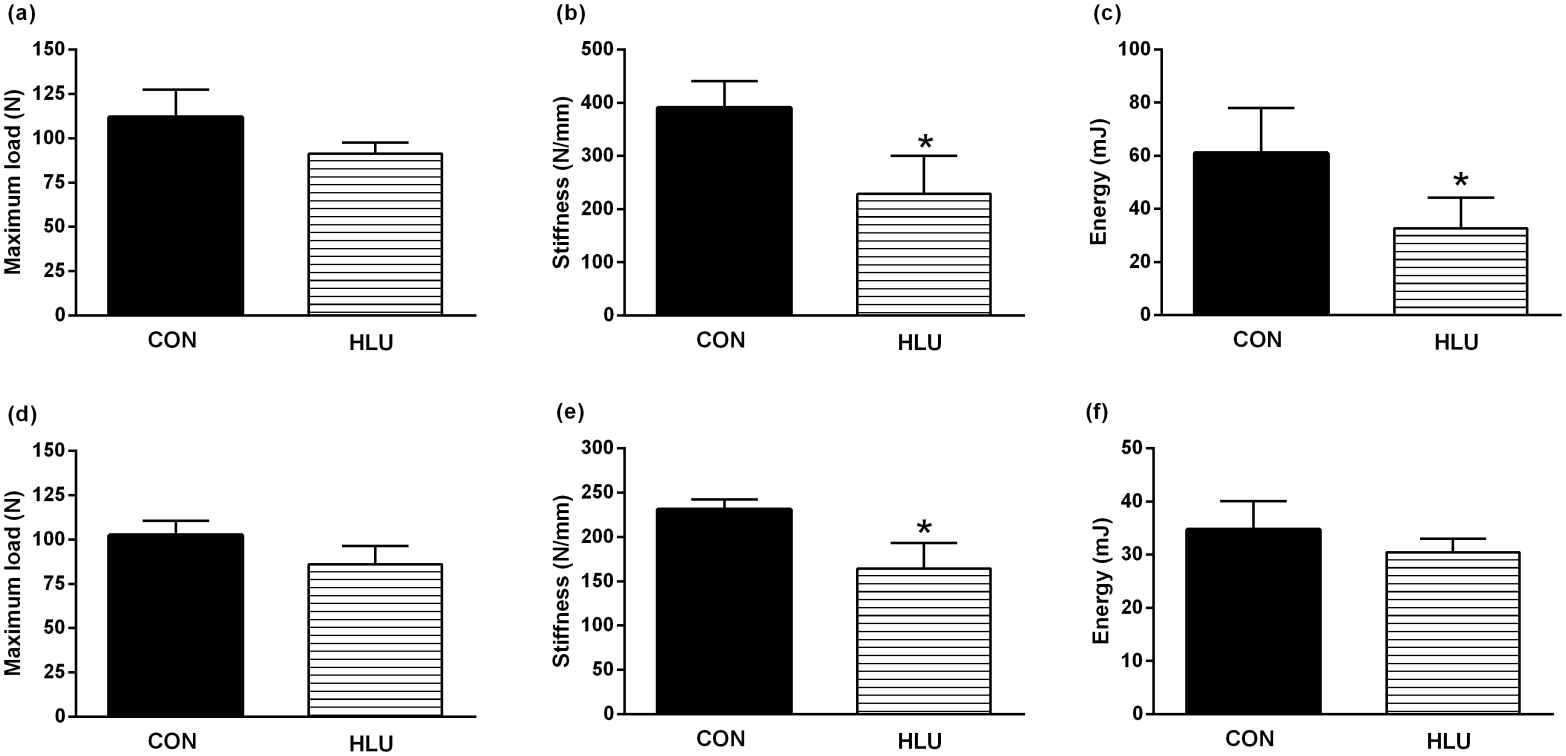
Biomechanical test parameters. *Ex vivo* maximum load (a, d), stiffness (b, e), and absorbed energy to maximum load (c, f) data of the femoral neck, assessed by the biomechanical compression bending testing *(top)* and data of the femoral midshaft assessed by the three-points bending testing *(below)*. Statistical analysis was performed with the Mann Whitney test to compare the hindlimb unloading (HLU) group to the control (CON) group * (*P* < 0.05).

The biomechanical properties of the femur were altered significantly after the disuse period (Fig 6). In the femoral neck, after 21days of disuse, the HLU group showed lower stiffness and energy absorbed to the maximum load, compared to the CON group (*P* = 0.002 and *P* = 0.031, respectively), but without significant differences on the maximum load (*P* >0.05) (Fig 6a–6c). In the femoral midshaft, there was lower stiffness in the HLU group compared with the CON group (*P* = 0.007) (Fig 6e). The maximum load and the energy absorbed to maximum load (Fig 6d–6f) were not different between the CON and HLU groups (*P* > 0.05).

### Cortical bone analysis by confocal laser Raman microspectroscopy

**Fig 7:**
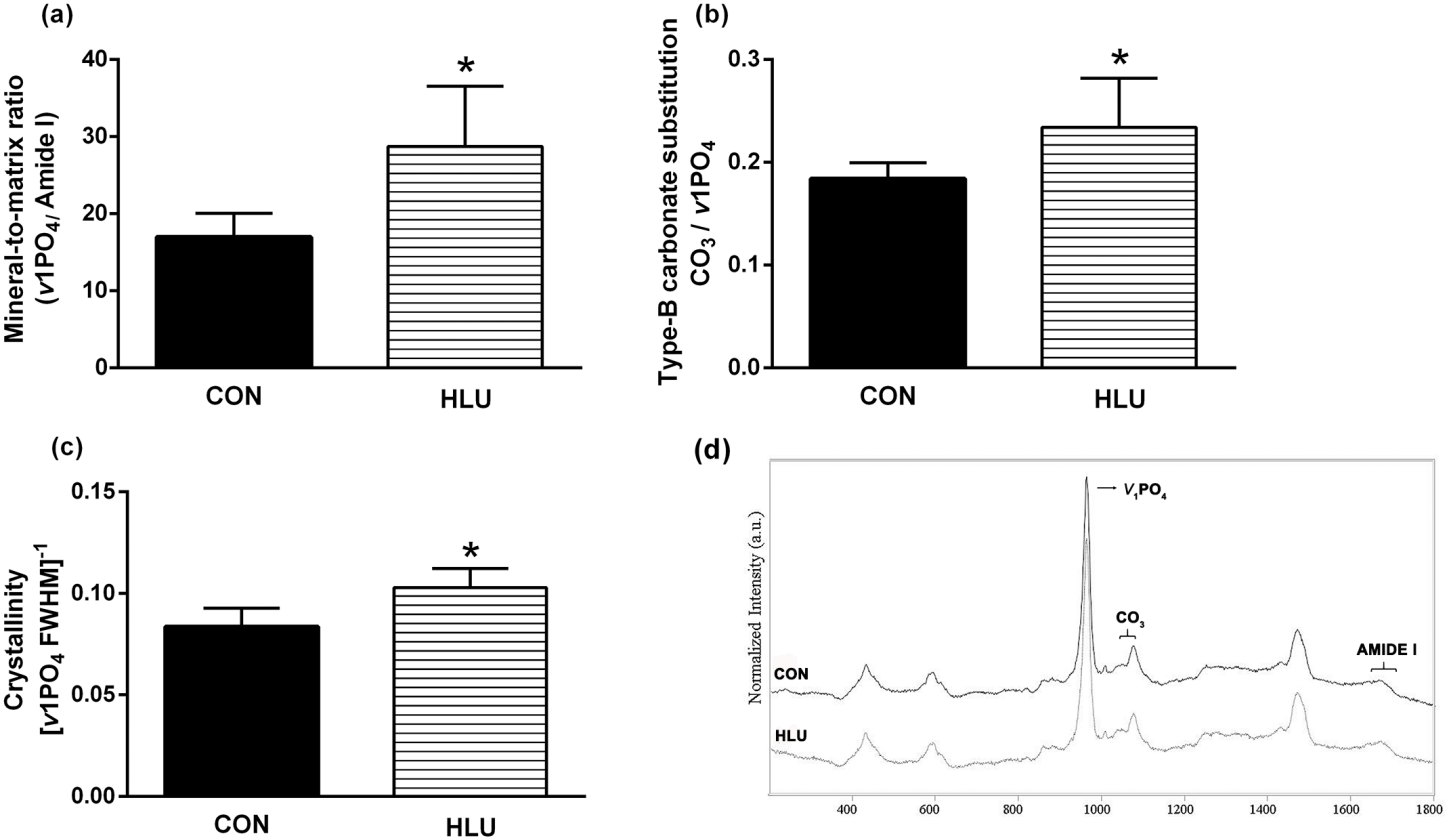
Raman microspectroscopy parameters obtained from the femoral midshaft. Mineral-to-collagen ratio (ν1PO4/Amide I) (a), type B carbonate substitution (CO3/ν1PO4) (b), and crystallinity (c). Representative femur spectra from both groups (d). Statistical analysis was performed with the Mann Whitney test to compare the hindlimb unloading (HLU) group to the control (CON) group * (*P* < 0.05).

We studied the matrix and mineral components in the cortical bone of the femur with confocal laser Raman microspectroscopy (Fig 7). Raman spectrum collected from CON and HLU animals demonstrated large changes in the physicochemical properties of cortical bone tissue after 21 days of disuse (Fig 7d). The mineral-to-matrix ratio (Fig 7a), as determined by the ν1PO4/Amide I was higher in the HLU group (*P* = 0.0001). Type B carbonate substitution (Fig 7b), as determined by CO3/*v*1 PO4, was highest after bone disuse (*P* = 0.012). Crystallinity (Fig 7c), as determined by the inverse of the full-width at half-maximum intensity of the *v*1PO4 peak, was higher in the HLU group compared with the CON group (*P* = 0.0001).

## Discussion

Our results demonstrated that disuse for 21 days, induced by HLU, in adult female Wistar rats (6 months) caused alteration of the bone microstructure and changes in physicochemical properties, which led to reductions in bone density and biomechanics deterioration. Therefore, this study shows the critical role of mechanical stimulation in maintaining the structure of the skeleton in female adults, and that disuse for even a few days, leads to microscopic changes in the structure of the bone matrix, which can increase the fracture risk.

The effects of HLU on the skeleton of young rats are well established [12, 15, 27]. However, few unloading studies have been performed in adult rats and especially in females. In the early 1990s [28] evaluated the effect of right hindlimb immobilization with an elastic bandage on the bone tissue of 9-month-old rats by means of single-photon absorptiometry and histomorphometry, and they observed that disuse reduced cortical bone mass and accelerated bone marrow expansion. The present results corroborate the previous data, since the lack of mechanical use by tail suspension has deteriorated the cortical tissue and increased the parameters for bone resorption. However, it is important to characterize changes in bone mineral and matrix tissue properties besides that bone microarchitecture, for enabling adequate and more specific analysis of bone loss after disuse.

Under physiological conditions, the process of bone remodeling continuously molds and repairs, maintaining the bone structural integrity and performs its function in mineral homeostasis. Disuse, caused by lack of mechanical stimulation, causes an uncoupling of bone remodeling, with a significant reduction in bone formation rate and a modest increase of bone resorption rate [12, 29]. This study was not intended to investigate the effects of HLU on the rate of bone resorption and formation; nevertheless, it is noticeable that after disuse there was decreased plasma alkaline phosphatase at the end of the experiment.

At the architectural level, of the features investigated, it is notable that after disuse, deterioration was most relevant in the cortical bone. In the trabecular analysis, the SMI showed significant change, contrary to some studies, which used male rats, and demonstrated major trabecular deterioration after disuse [16, 30]. However, in females, where estrogen receptor α (ERα) is one of the key components for bone metabolism, our result seems understandable. One study deleted the estrogen receptor from the monocyte/macrophage cell lineage in mice and found a 2-fold increase in the number of osteoclasts in cancellous bone, showing the importance of estrogen to trabecular protection [31]. Maria Almeida et al. [32] deleted ER α in different stages of cell lineage differentiation of osteoblasts and found that the periosteal apposition allowed the participation of ERα in the transduction of signals acting on mechanical activation of the Wnt pathway without the need of estrogen. Others [33] investigated bone adaptation during simulated weightlessness between males and females. It had been observed that, in the Wistar male rats, there was a dramatic reduction in trabecular tissue, while in females the deterioration was less dramatic. It appears that the lack of mechanical stimulation mainly changes the cortical compartment and that the estrogen still circulating in the organism, may have acted as a protector of the trabecular tissue.

The structural and geometric properties of cortical bone are of paramount importance for fracture risk prediction [9], because 70% of all appendicular bone loss is in cortical bone. In this study, we demonstrated that the HLU group had an increased medullary volume and decreased cortex fraction, and showed that cortical bone is prominently affected by unloading in skeletally mature female rats. Furthermore, we demonstrated that 21 days of disuse resulted in increased Ct.Po (%), making bones fragile and susceptible to fracture. The trabecular and cortical microstructure, in response to reduced weight-bearing in humans [34] showed a dramatic increase of the porosity during a6-week disuse period, with significant changes in biomechanics, including decreased stiffness and estimated failure load. A recent study showed that Ct.Po (%) is a risk factor for fracture, independent of aBMD and assessment by the Fracture Risk Assessment Tool, and improves identification of women with fractures [8].

To the best of our knowledge, this study is the first to quantify and measure the amount of the chemical substances present in the bone tissue after HLU or any method that induces disuse. Raman spectroscopy is emerging as an important complement to traditional methods of bone assessment, providing information about both the matrix and the mineral composition, which are directly related to the quality and mechanical competence of bone tissue [35]. It was observed that the physicochemical properties of bone tissue were changed such that the collagen matrix was more mineralized, there were more carbonate ions occupying the stoichiometric phosphate locations, and higher crystallinity compared with control group. A mineral to collagen-increasing intensity ratio (ν1PO4/amide I) indicates more mineralized collagen; this change in composition and loss of heterogeneity reflects the persistence of old bone and decreased new bone formation, and this matrix can be associated with decreased bone ductility due to reduced mobility of collagen fibers [36]. Some phosphate ions can be replaced with carbonate ions classified as B-type carbonate apatite. When the carbonate is present in the hydroxyapatite crystal, this makes it an impure mineral, and this substitution changes the shape of mineral crystals, deteriorates the symmetry, and creates lattice vacancies within the crystal lattice [37], which may cause damage to the mechanical properties [38]. In this study, it was also observed that disuse provided a higher crystallinity compared to the control group. The presence of the mineral crystal size is associated with decreased formation of new smaller crystals and the increase of crystallinity results in a decrease in yield stress and strain at rupture [39].

As expected, along with increased crystal size, presence of impure minerals, and increased mineralization, the bone showed that bone deformation capacity and absorbed energy were altered after disuse. The fragility of the structural organization altered the biomechanical properties, which caused not only lower strength, but also toughening of the mechanisms, where the plastic nature of the tissue is important. In recent years, studies have shown that the structural properties and cortical material composition demonstrate a critical role in the skeletal load-bearing performance [40, 41], and an understanding of these microstructural changes may have clinical relevance in the development of therapeutic approaches to reduce the possible incidence of osteoporosis and fracture after a period without mechanical stimulation. Since the Raman methods of bone assessment provide this information, they have the potential to assess fracture risk.

Like any animal model, there are limitations compared to human clinical evidence; however, these models can simulate the changes of bone metabolism similar to humans, and enable us to avoid more invasive analysis. However, the findings that were introduced here contribute to elucidating the etiology of the deterioration in bone induced by disuse, where lack of mechanical stimulation in the skeleton of female adults, despite having had estrogen circulating in their bodies, caused alteration of the microstructure and changes in physicochemical properties, leading to a decrease in bone density and deterioration of biomechanics.

## Supporting information

S1 Fig Animal in the suspension cage with hindlimb unloading. (BMP)
