# Supplementary figures and images for: Model of hindlimb unloading in adult female rats: Characterizing bone physicochemical, microstructural, and biomechanical properties
Source: PLoS One. 2017 Dec 11;12(12):e0189121. doi: 10.1371/journal.pone.0189121 (PMC5724829; doi:10.1371/journal.pone.0189121)

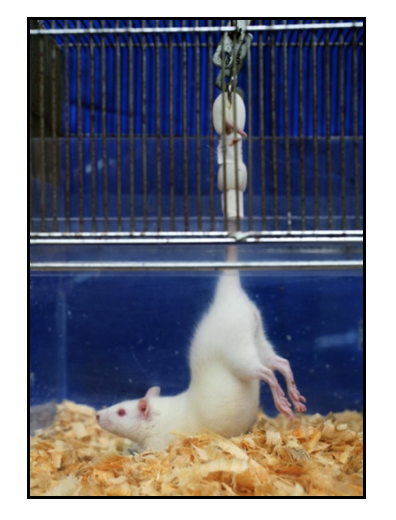

Supplement: S1 Fig — (BMP) [file pone.0189121.s001.bmp]
